# Preparation and Characterization of Magnetic Solid Lipid Nanoparticles as a Targeted Drug Delivery System for Doxorubicin

**DOI:** 10.34172/apb.2023.033

**Published:** 2022-01-03

**Authors:** Abbas Soltani, Parvaneh Pakravan

**Affiliations:** Department of Chemistry, Zanjan Branch, Islamic Azad University, Zanjan, Iran.

**Keywords:** Doxorubicin, Magnetic Solid Lipid Nanoparticle, Target drug delivery, Stearic Acid

## Abstract

***Purpose:*** In the present study, we investigated the magnetic solid lipid nanoparticles (mSLNs) for targeted delivery of doxorubicin (DOX) into breast cancer cells.

***Methods:*** The synthesis of iron oxide nanoparticles was carried out by co-precipitation of a ferrous and ferric aqueous solution with the addition of a base; moreover, during precipitation process, the magnetite nanoparticles should be coated with stearic acid (SA) and tripalmitin (TPG). An emulsification dispersion-ultrasonic method was employed to prepare DOX loaded mSLNs. Fourier transforms infrared spectroscopy, vibrating sample magnetometer, and photon correlation spectroscopy (PCS) were used to characterize the subsequently prepared nanoparticles. In addition, the antitumor efficacy of particles was evaluated on MCF-7 cancer cell lines.

***Results:*** The findings showed that entrapment efficiency values for solid lipid and magnetic SLNs were 87±4.5% and 53.7±3.5%, respectively. PCS investigations showed that particle size increased with magnetic loading in the prepared NPs. In vitro drug release of DOX-loaded SLN and DOX-loaded mSLN in phosphate buffer saline (pH=7.4) showed that the amount of drug released approached 60% and 80%, respectively after 96 h of incubation. The electrostatic interactions between magnetite and drug had little effect on the release characteristics of the drug. The higher toxicity of DOX as nanoparticles compared to free drug was inferred from *in vitro* cytotoxicity.

***Conclusion:*** DOX encapsulated magnetic SLNs can act as a suitable and promising candidate for controlled and targeted therapy for cancer.

## Introduction

**Scheme 1:**
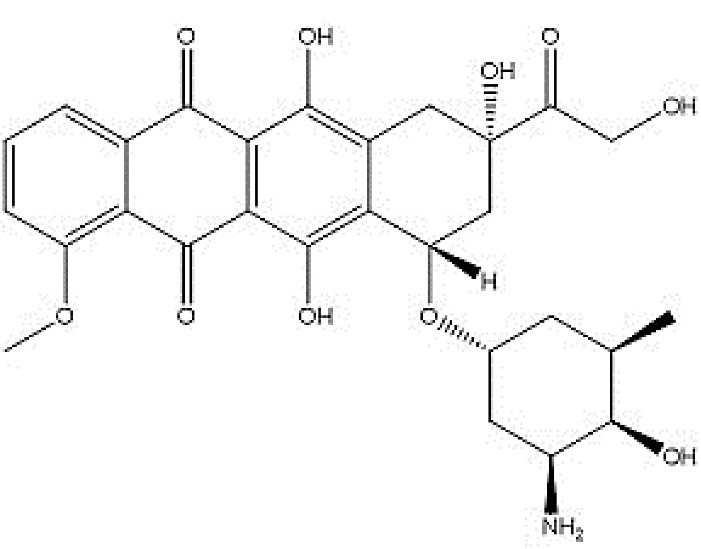
Doxorubicin chemical structure.

One of the most noticeable forms of chemotherapy is cytotoxic agents. Cytotoxic drugs can be described as a different class of therapeutic agent. Interestingly, cancer is treated by this agent primarily by being toxic to rapidly dividing and growing cells.^1^ As a chemotherapeutic agent, doxorubicin (DOX, Scheme 1) is one of the leading treatments for early and advanced breast cancers. However, regardless of its effectiveness, it leads to a set of undesirable side effects, especially irreversible cardiotoxicity and reversible nephrotoxicity; these side effects have resulted in the development of many different FDA approved carriers. The antitumor effects of DOX are less than or equal to the approved DOX-loaded nano-carriers. These carriers *improve* the *cancer* treatment by increasing tumor accumulation of these carriers through enhanced vascular permeability and retention effect. However, an important problem with existing drug delivery preparations of DOX is the lack of efficacy against tumors that exhibit multidrug resistance. As a matter of choice, since DOX-containing NPs are endocytized and located near the perinuclear membrane, the cellular membrane efflux mechanisms may be less effective in reducing DOX levels compared to free DOX that passively enters the cell.^2^ Whether in combination with other forms of chemotherapy or individually, DOX is an acknowledged first-line therapy for a variety of cancers such as lung, ovarian, breast, and bladder.^3^ Consequently, increasing the absorption and penetration at specific DOX target tissues of DOX by developing a drug delivery system and improving anticancer activity is of substantial importance.

Since the early 1990s, solid lipid nanoparticles (SLNs) have been defined as a potential system for drug delivery to predictable carriers.^4^ SLNs have been proposed as novel colloidal drug delivery carriers composed of biocompatible lipid matrix that dispersed in an aqueous surfactant phase as a stabilizing agent.^5^ The advantages of SLN in drug delivery over common carriers include low toxicity, suitable biocompatibility, and targeting effect on the brain.^6,7^ SLNs were administered through rectal, parenteral, oral, dermal, and ocular, and pulmonary routes.^8,9^ Given the significant benefits of SLNs compared to alternative carriers, researches on SLNs in targeted drug delivery is gaining more attention.^10,11^

The application of nanodelivery systems for payload release under physiological conditions is known as passive delivery and can be specific for tumors.^12,13^ The drug delivery system in treatment of locoregional cancer has been targeting the magnetic drug. It is noteworthy that the most common use of delivering chemotherapeutic drugs are coated magnetic particles.^14^

Commonly used as magnetic response agents that have super-paramagnetic properties, iron oxide NPs can also be controlled from outside the body using a magnetic field.^15^ It should be noted that magnetic nanoparticles (mNPs) are biologically inactive; however, mNPs are detached from the blood circulation via reticuloendothelial system or absorbed through plasma proteins following intravenous injection.^16^ Adsorption of plasma protein is decreased by surface modification with mNPs coating through proteins, lipids, polymers, and polysaccharides; in such a case, the mNPs remain in the bloodstream for a longer.^17,18^ A number of applications have been discovered in cancer biology including drug delivery, therapy, and targeting for iron oxide NPs.^19,20^

As a new and modern targeting drug delivery system,^21^ mSLNs have demonstrated significant use in cancer therapy over the past few years.^22,23^ Consequently, while the dosage and toxicity were reduced for chemotherapy drugs, the reliability, validity, and patient compliance to these drugs increase dramatically.^24^ In recent years, we have seen the design of vehicles of targeted drug delivery to formulate core/shell spheroidal nanoparticles; NPs consist of Fe_3_O_4_ cores coated with carrageenan,^25^ or with siliceous shells enriched with starch polymers,^25^ silica shells,^26^ chitosan,^27^ and alginate.^28^

Investigations carried out in this study lay out the optimization and preparation of loading DOX into SLN-coated iron oxide NP carriers; in addition, the release profile related to these carriers was studied and their effectiveness for cytotoxicity were evaluated. Accordingly, DOX enclosed magnetic loaded SLNs were arranged and identified in terms of size, uniformity, and magnetic property. Formulation tests were conducted for MCF-7 cell cytotoxicity and *in vitro* cellular uptake and then compared to its free form (DOX).

## Materials and Methods

###  Materials

 Materials were purchased from Merck (Darmstadt, Germany) included Fe (II) chloride tetrahydrate (99%), Fe (III) chloride hexahydrate (97%), and stearic acid (SA). Furthermore, 3-(4, 5-dimethyl-thiazol-2yl)-2, 5-diphenyltetrazoliumbromide (MTT) was purchased from Sigma-Aldrich (St Louis, MO, USA). Ultimately, tripalmitin (TPG) was purchased from Alfa Aesar Co. (Germany). It is important to note that all other reagents were the highest available grade and were provided from commercial sources.

### Synthesis of Fe_3_O_4_ mNPs and preparation of mSLNs

Reaction between FeCl_2_.4H_2_O and FeCl_3_.H_2_O in 1:2 molar ratio produced iron oxide mNPs in distilled deionized water (20 mL) by mixing at 250 rpm for 15 minutes under N_2_ atmosphere, at 60°C. Next, the solution was supplemented with 100 mL of ammonium hydroxide (8 M). Subsequently, the color of the mixture immediately changed from yellow to black. Then, the solution was mixed again at 400 rpm for an additional 10 minutes.^29^ Finally, 5% NH_4_OH was used to wash the precipitated mNPs several times.

Preparation of SLNs and mSLNs was carried out based on the previously described micro-emulsion process^30,31^ with minor changes. Micro-emulsions were prepared using oil, water, and surfactant. Oil droplets were stabilized at the liquid-liquid boundary by adsorption of the stabilizing layer. To obtain SLN, micro-emulsion (O/W) technique was employed via TPG and SA as the lipid core. After consuming the hot oily phase with DOX (5 mg), a double-distilled water aqueous solution (10 mL) containing polysorbate 80 was added to DOX and the melted lipid.

Before mixture sonication using a probe sonicator (60 Watt) for 2 minutes at constant temperature, the sample was agitated at 1000 rpm for 30 minutes. The hot emulsion was then separated in icy distilled water (2-5ºC) in the emulsion to obtain the SLNs. The emulsion specifications are as follows: Water ratio 1:5 (additional speed: 2 mL/min, mixing speed: 500 rpm and a syringe needle gauge: 25). A probe sonicator (60 Watt) was then employed to further process the SLNs through ultra-sonication in an ice bath for 2 minutes. For an additional hour, the SLNs were dialyzed using cellulose acetate dialysis membrane (molecular weight cutoff [MWCO] of 12 kDa) against distilled water in order to remove unentrapped surfactants and DOX. The same approach was adopted to prepare mSLNs and then added mNPs to the oil phase with DOX. During the preparation of mSLNs, the samples were degassed via N_2_ at predetermined intervals. Entrapment efficiency (EE) was calculated using dialyzed media. Finally, the samples were freeze-dried for further experiments and evaluations.

###  In vitro Assays

####  Determining encapsulation efficiency 

Spectrophotometric determination at 498 nm using a spectrophotometer was employed to indicate the incorporating DOX (EE) percentage (Shimadzu, Japan). In order to perform this experiment, the acquired mSLNs were sedimented by magnet force followed by measuring the amount of drug in the supernatant. The EE percentage of the drug in the NPs was calculated using the following equation (1)^32,33^:


(1) $$\%EE=\frac{DOX_{0}-DOX_{supernatant}}{DOX_{0}}\times 100$$

Where DOX_0_ is the initial amount of drug in the mSLN–DOX conjugate and in the production of NPs and DOX_supernatant_ is the amount of DOX which is detected after centrifugation of NPs in the supernatant.

####  Characterization of synthesized magnetic nanoparticles

Photon correlation spectroscopy (PCS) was used to measure the particle size and the polydispersity index (PDI) of the NPs (Malvern Zetasizer ZS; Malvern, UK). Prior to measurement, the dried powder samples were moderately sonicated and suspended in ultra-purified water. Next, the PDI and the mean diameter of the resulted in homogeneous suspensions were evaluated. The morphology and particle size of the nanoparticles were observed using a transmission electron microscope (TEM, JEOL JEM-2000 EX II). A copper grid was prepared with carbon coating and an aqueous dispersion of particles was dropped-cast onto it; next, the grid was air-dried at room temperature before loading into the microscope. Fourier transform infrared spectroscopy (FTIR) DOX, mNP and mSLN were recorded in an infrared spectrophotometer (Perkin Elmer Spectrum BX, USA). A mixture was then prepared containing the samples as well as finely ground potassium bromide (KBr) prior to compression under considerably high pressure to create a small disk of approximately 1 cm in diameter. The resulting transparent disks were then scanned with a wavelength of 4000–400 cm^-1^. The magnetization measurements of the prepared nanoparticles on a vibrating scanning magnetometry (VSM) (HH-15, Nanjing University Instrument Plant, China) were demonstrated the M vs. H curve of the NPs with a field applied in the range of -15000 to 15000 Oe at room temperature. After placing the samples in vials (2 mL), they were measured as dispersion aqueous.

###  In vitro release evaluation

 Dialysis membrane method was used to perform DOX release of the formulation. Briefly, freeze-dried samples (5 mg) were first dispersed in PBS (1 mL) and then transferred to cellulose acetate dialysis bags (MWCO: 12,000 Da). Next, the dialysis bag was moved to a glass vessel containing PBS (25 mL, 0.1 M, incubated at 37°C, stirring 250 rpm, pH 7.4). The release medium (1 mL) would be picked up at particular intervals, followed by an equal amount of fresh PBS added to the vessel. In order to measure the sample absorbance, a UV-Vis spectrophotometer was used at 480 nm. Ultimately, the DOX concentration was calculated from the drug calibration curve.

#### Cytotoxicity studies

MTT assay in MCF-7 cell line, grown in RPMI1640 culture media (Gibco), was used to evaluate the anti-proliferative activity of DOX-NPs and DOX at various concentrations.^34^ In addition, MCF-7 cells were supplemented with 1% non-essential amino acid (NEAA, Sigma), 10% fetal bovine serum (FBS, Gibco), 100 IU/mL penicillin (Sigma), 2mM L-glutamine (Sigma), and 100 µg/mL streptomycin (Sigma) in T-25 cm^2^ tissue culture flasks. 5% CO_2_ was selected as the medium in which the cultures were incubated every 2 days at 37°C. Cell cultures were trypsinated using trypsin-EDTA 0.25% (Sigma) as they reached 70-80% confluency. Next, cell cultures were sub-cultured at 1 × 10^4^ cells/well density inside 24-well culture plates. Cells were washed using PBS (pH 7.4) one day after implantation. Three groups were identified as follows:

- Group I: incubated with DOX;
- Group II: incubated with DOX-SLN;
- Group III: incubated with DOX-mSLN.

Moreover, each group entailed eight treatments which included (1) Control: 0.0 μg; (2) Treatment 1: 100 nM; (3) Treatment 2: 250 nM; (4) Treatment 3: 500 nM; (5) Treatment 4: 1000 nM; (6) Treatment 5: 2000 nM; and (7) Treatment 6: 4000 nM. In the next step, the cells were moved to the incubator with 5% CO_2_ at 37°C. Culture medium (model RPMI1640) containing 0.2% BSA was used to culture the cells. The MTT assay was then measured to quantify cells viabilities. This experiment was carried out by loading 15 × 10^3^ cells into a 96-well plate followed by adding RPMI1640 media (200 μL) containing 0.2% BSA. After incubation for a full day, the wells were supplemented with 200 μL of treatments media as previously explained. Next, the cells were incubated separately for one day in a diverse treatment environment. MTT test was performed after incubation. In this experiment, supernatant was separated from each well before adding 50 μL of MTT solution (5 mg/mL) to each well and then incubated for 180 minutes. After removing the supernatant from each well once again, dimethyl sulfoxide (100 μL) was added to dissolve the formazan crystals at room temperature for half an hour. An enzyme-linked immunosorbent assay (ELISA) reader at 570 and 630 nm was used to measure the optical density of each well.

 The following formula was used to calculate cells viabilities for each concentration:


(2) $$Cell\;viability\;\%=A_{treated\;cells}/A_{untreated\;cells}\times 100$$

## Results and Discussion

 In this study, the magnetic, morphological, and structural properties of uncoated and solid lipid coated iron oxide nanoparticles were presented. Particle characterization was applied by TEM, FTIR, and VSM. In addition, release studies of solid lipid coated iron oxide nanoparticles as well as DOX loading were provided. There have been numerous reports of various synthetic routes for mNP synthesis. One of the oldest approaches to mNP preparation is called, ‘wet precipitation’. Iron oxide is formed as a fine suspension with small particle size (~5 nm) by controlling the pH of an iron salt solution. Interestingly, this simple mNP preparation method does not require any special features.

The preparation of mixed oxide particles can be done by co-precipitation as well with a stoichiometric solution of two metal ions. For example, magnetite is noteworthy; this material can be prepared by supplementing a mixture of Fe^2+^ and Fe^3+^ solution with base according to the following equation^35^:


$$Fe2+\left(aq.\right)\;+Fe3+\left(aq.\right)\;+8OH-\left(aq.\right)\;\to \;Fe3O4\;\left(s\right)+4H2O\;\left(I\right)$$

Chemical co-precipitation of ferric and ferrous salts in alkaline medium was used to prepare iron oxide mNPs. Importantly, these particles were probe-sonicated before magnetic loading; to avoid mNP aggregation at this stage, lipid was used as the dispersion agent while the mNPs and drug were coated. Fabrication of mSLNs required optimum conditions which involved a homogenizer speed of 30 000 rpm and a weight of TPG: SA as core lipid 2:3. Table 1 presents the preparation condition. According to the literatures, 5 mg of mNPs and DOX were selected in all experiments.^10,36^ Nonetheless, PCS is not a proper method for particle investigation as a result of the aggregation of NPs in the suspension due to the super-paramagnetic behaviour of formulations.

**Table 1:** Optimum nanoparticles preparation

**TPG (mg)**	**SA (mg)**	**Tween 80 (mg)**	**Hot microemulsion: cold water (ml)**	**Drug (mg)**	**mNPs**	**Size (nm)**	**PDI**	**EE%**
25	25	15	5 : 50	-	-	389	0.559	-
20	30	15	5 : 50	-	-	399	0.448	-
30	20	15	5 : 50	-	-	263	0.617	-
20	30	7.5	5 : 50	-	-	237	0.324	-
20	30	7.5	10 : 50	-	-	133	0.34	-
20	30	7.5	10 : 50	5	-	153	0.406	87
20	30	7.5	10 : 50	5	5	194	0.68	53.7

**Figure 1:**
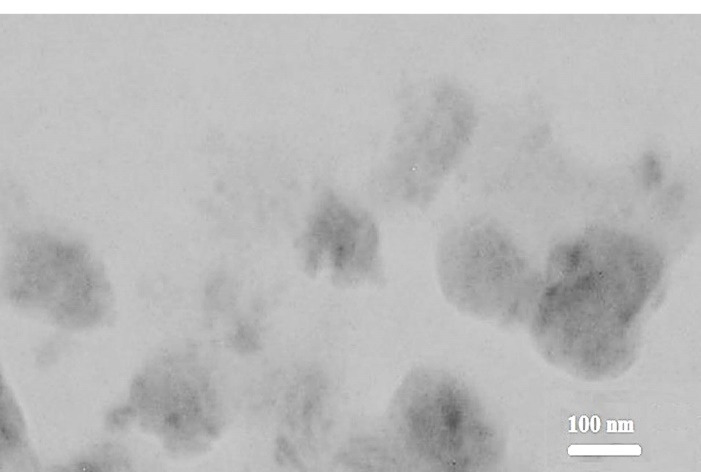
Transmission electron micrograph of nanoparticles.

As shown in the TEM micrograph illustrated by Figure 1, the lining between the black region (magnetite) and a lipid layer (surrounded by a gray region) of mSLNs represents uniform particle morphology. Moreover, NPs with marginally deformed round contours and a diameter of approximately 200 nm was witnessed. Accordingly, several Fe_3_O_4_ mNPs were entrapped and dispersed in the SLN, and the average diameter for formulation of the TEM was estimated to be less than 10 nm. Based on the TEM micrographs, a number of Fe_3_O_4_ mNPs (with particle sizes of less than 10 nm) were entrapped and dispersed within the SLN. Here, the particle diameters suggested that the synthesized mNP were super-paramagnetic.^37^

 A suitable technique for the structure confirmation of SLNs, mNPs and conjugating drug through solid lipid coated mNPs is FTIR.

**Figure 2:**
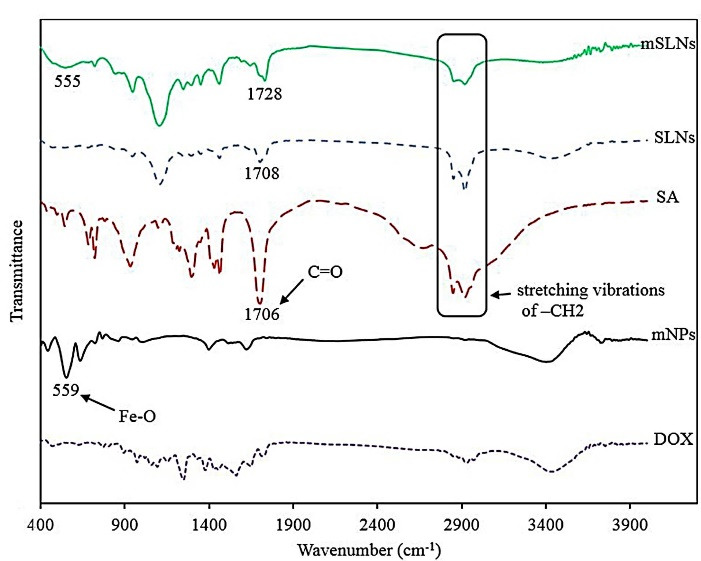
The FT-IR spectra of samples including mNPs, SA, DOX, SLN, mSLNs from 400 to 4000 cm^-1^.

The standard purpose in preparing nanoparticle is to fully load the drugs into the carrier; therefore, conducting investigations on this issue is of substantial importance.^38,39^ For this purpose, the FTIR spectrum of mNPs, SA, SLNs, mSLNs, and pure drug were acquired as demonstrated in Figure 2. The presence of Fe_3_O_4_ mNPs should be confirmed for mSLNs by observing a robust peak at approximately 550 cm^-1^ for the Fe-O bond.^40^ In case of uncoated iron oxide particles, the bands at 3394 cm^−1^ and 1620 cm^−1^ are assigned to stretching (ν) and bending (δ) vibrations, respectively.^41^ As a result, water is adsorbed on the surface of iron oxide nanoparticles. There is a correspondence between the band witnessed at 611 cm^−1^ and the stretching vibrations of M_Th_–O–M_Oh_; in this case, there is a correspondence between M_Th_ and M_Oh_, and iron occupying the tetrahedral and octahedral positions, respectively. Observations of SLN-coated iron oxide nanoparticles included an alcoholic O–H stretching band at 3410 cm^−1^ and M_Th_–O–M_Oh_ stretching band at 606 cm^−1^. The binding of SLNs to iron oxide nanoparticles were approved by the following observations: correspondence between additional bands at 2912 cm^−1^ and C–H stretching vibrations; at 1416 cm^−1^ and C–C stretching vibration; at 1092 cm^−1^ attributable to the M–O–C (M = Fe) bond; and at 850 cm^−1^ corresponding to CH_2_ rocking in solid lipid coated iron oxide nanoparticles.

**Figure 3:**
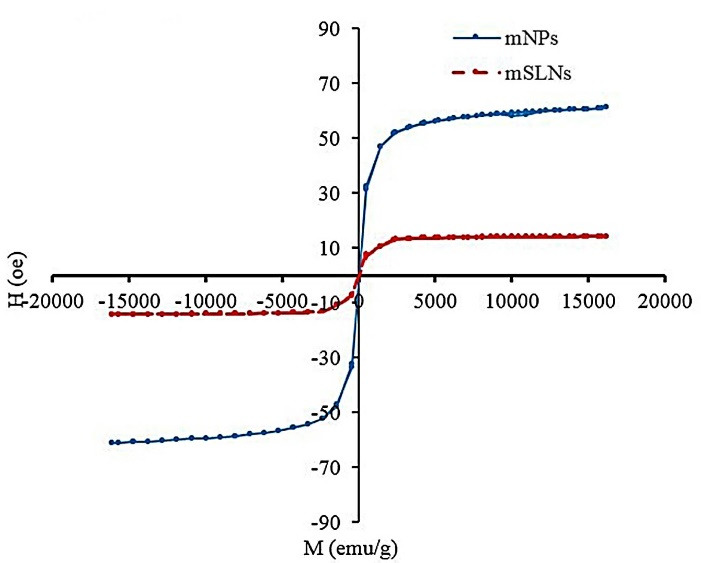
The VSM spectrum of mSLN, mNPs. M versus H curve of mSLN and mNP indicate the single domain superparamagnetic properties of mNPs.

Figure 3 showed the magnetic properties of mSLNs. The coercive force (applied field that decreases magnetization to 0) and remanence (residue magnetization) were 0 of the magnetization curve; furthermore, no magnetic hysteresis loop was observed to characterize the super-paramagnetic behavior of mNPs. For magnetic materials with a size of below 10 nm, this is fairly normal.^42^ According to these findings, the monodispersity of mNPs in SLNs were proven. Superparamagnetism refers to the responsiveness in an applied magnetic field with no need to maintain any magnetism after the magnetic field is removed. Consequently, it is a particularly significant property required for magnetic targeted carriers.^43,44^ There were the saturated magnetizations of 16.4 and 57.5 emu/g correspond to mSLNs and mNPs, respectively. The magnetization value ​​of mSLNs was significantly lower compared to pure magnetite nanoparticles. This is the result of the lower weight of mNPs in mSLNs compared to pure mNPs. However, when Fe_3_O_4_ mNP is covered, then the saturation magnetization would be reduced as well.^45^

###  In vitro drug release study

**Figure 4:**
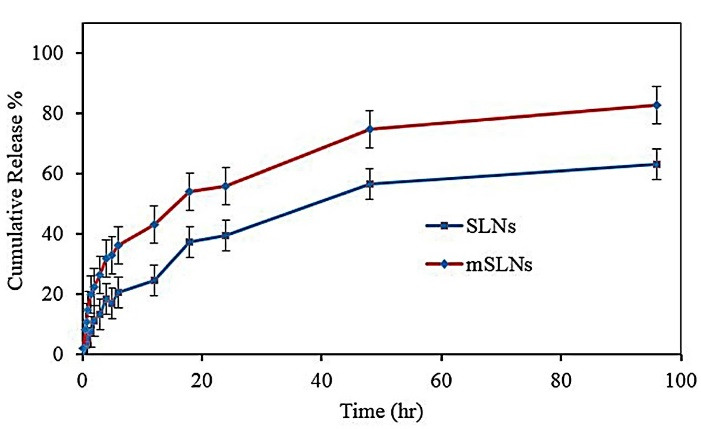
In vitro drug release of NPs in sink condition (PBS: pH 7.4, 0.1 M).

Due to their lyophilic phenomenon, SLNs showed more controlled release compared to other drug carriers.^46^ However, their modifications led to variations in drug release.^47^ Figure 4 showed the percentage of cumulative release with a summary of the *in vitro* release profile of DOX in both SLNs and mSLNs sink conditions. During the first hour of release, the initial burst release was significant for formulations. The reason behind this initial burst can be attributed to a portion of drug adsorbed on the surface of NPs and/or the presence of drug in vicinity of the NPs’ surface. The delayed part in both formulations was caused by the release of drug molecules through the lyophilic core matrix and its gradual exiting to the medium of dissolution. For conventional SLNs, this condition is clearer; accordingly, for this formulation, approximately 60% of DOX was released in 96 hours. Increasing this value to more than 80% for mSLNs may be due to the structural effects of mNPs on the obtained mSLNs.

###  In vitro cytotoxicity

Following a full day of exposure to DOX at various concentrations, MTT assay was used on MCF-7 cell lines to evaluate the cytotoxic activity of DOX loaded nanoparticles.^48^ After generating dose-effect curves, drug sensitivity to targeted and un-targeted SLNs and free DOX were expressed as drug concentration leading to 50% growth inhibition (IC_50_). DOX -NPs involved a variety of toxicity; for instance, compared to conventional SLNs, the targeted solid lipid nanoparticle were significantly toxic against the cell line.

**Figure 5:**
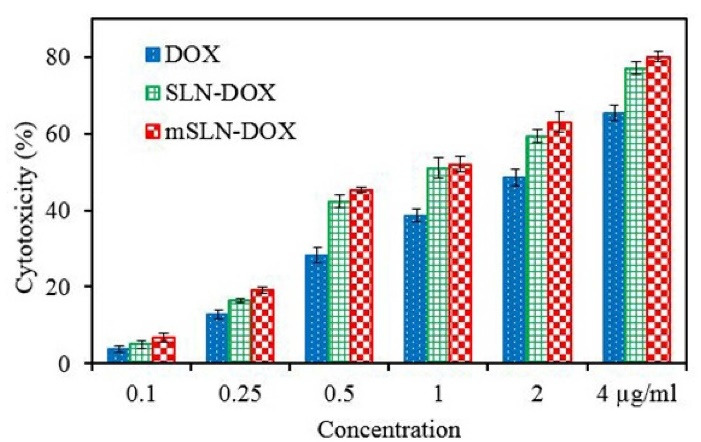
The cytotoxic effect on the MCF-7 cell line after treatment with different concentrations of DOX, drug loaded mSLNs and SLNs.

Base on the cell cytotoxicity graph (Figure 5), there was no detectable difference between SLN-DOX and mSLN-DOX complex. The use of mNPs as a drug or gene delivery can contribute to the effectiveness of cancer treatment in a variety of ways. One of the advantages of using mNPs over non-mNPs is that their magnetic behavior allows MRI to observe and quantify the biodistribution, resulting in optimal doses for the treatment of cancer. Second, targeting tumors with mNPs can overcome some additional barriers to more effective treatment of the cancer, such as inadequate bloodstream penetration of certain therapeutic agents within the tumor. Third, targeting tumors with magnetically induced nanoparticles provides site specificity and thus provides treatment options, leading to fewer side effects and lower treatment costs. And finally, the use of the magnetic field as the driving force represents an aggressive therapeutic approach.^49^ Overall, *in vitro* investigation into cytotoxicity showed the positive effect of drug encapsulation on anti-cancer drug effects.

## Conclusion

The experiments were performed in this study involved the successful formulation of DOX into SLN magnetic NPs with a sufficiently small particle size to be suitable for targeting delivery vehicles. For SLNs and mSLNs, entrapment efficiency values were obtained equal to 87 ± 4.5% and 53.7 ± 3.5%, respectively. The saturation magnetization for the mSLNs and mNPs was observed to be 16.4 and 57.5 emu/g, respectively. It is generally accepted that coating with SLN tends to cause a decrease in saturation magnetization due to the increase in total mass. In vitro cytotoxicity studies showed the beneficial effect of drug encapsulation on the effect of anticancer drugs. The high cytotoxicity of optimized mSLNs was shown toward MCF-7 cells. Increasing the release in *in vitro* to more than 80% for mSLNs may be due to the structural effects of mNPs on mSLNs. A combination of SLNs and mNPs as the targeted drug delivery system could be valuable for applications including drug targeting and controlled drug release. Future studies will be necessary to know the applicability of this double framework against breast cancer.

## Acknowledgments

 The authors thank the Islamic Azad University of Zanjan for partial financial support of this work.

## Competing Interests

 The authors have no conflict of interest.

## Ethical Approval

 Not applicable.
